# Fracture load analysis of telescopic overdentures fabricated from PEEK and zirconia

**DOI:** 10.6026/973206300213973

**Published:** 2025-10-31

**Authors:** Anil Paul Melit, Miriam Mathew, Deepika Rayan Pai, Ipsita Saikia, Kavya M.J, Aravind A

**Affiliations:** 1 Restorative Dentist, My Dentist, Ludlow, UK; 2 Department of Prosthodontics, Crown & Bridge, Indira Gandhi Institute of Dental Sciences, Nellikuzhi PO, Kothamangalam, Ernakulam, Kerala, India; 3 Department of Prosthodontics, Crown & Bridge, Century International Institute of Dental Sciences and Research Centre, Poinachi, Kasargod, Kerala, India; 4 Department of Prosthodontics, Crown & Bridge, Aesthetic Dentistry and Oral Implantology, Government Dental College Silchar, Cachar, Assam, India; 5 Department of Public Health Dentistry, Sharavathi Dental College and Hospital, Shivamogga, Karnataka, India; 6 Department of Public Health Dentistry, Travancore Dental College, Kollam, Thazhuthala, Kerala, India

**Keywords:** Telescopic overdentures, polyetheretherketone (PEEK), Zirconia, fracture load, prosthodontics, universal testing machine, CAD/CAM dentistry

## Abstract

Edentulous patients often face challenges with denture retention and stability, making telescopic overdentures a preferred prosthetic option. The choice of material critically affects the strength and longevity of these restorations. Therefore, it is of interest to compare the fracture load resistance of telescopic overdentures fabricated with PEEK and zirconia copings after thermocycling. Zirconia demonstrated significantly higher fracture load values (1298.4 ± 110.7 N) than PEEK (845.6 ± 92.3 N, p = 0.002), though PEEK showed more favorable, repairable failure patterns. While zirconia provides superior fracture resistance, PEEK remains advantageous for its light weight, flexibility and ease of repair, emphasizing the need for material selection based on clinical demands.

## Background:

Telescopic overdentures represent a significant advancement in prosthodontic rehabilitation, offering enhanced retention, stability and distribution of occlusal forces for edentulous patients [1]. This prosthetic design consists of primary copings cemented to abutment teeth and secondary copings incorporated into the denture base, creating a precision attachment system that facilitates both function and maintenance [2]. The success of telescopic overdentures largely depends on the mechanical properties of the materials used in their fabrication, which must withstand masticatory forces while maintaining long-term clinical performance [3]. Traditional materials for telescopic crowns have included noble and base metal alloys, which offer excellent mechanical properties but present challenges related to esthetics, weight and potential allergic reactions [4]. In recent years, alternative materials such as polyetheretherketone (PEEK) and zirconia have gained popularity in prosthodontics due to their favorable biocompatibility, adequate strength and improved esthetic qualities [5]. PEEK, a high-performance polymer, has been increasingly utilized in dental applications due to its favorable mechanical properties, low weight, elastic modulus similar to bone and reduced stress transmission to abutment teeth [6]. Studies have demonstrated that PEEK exhibits good wear resistance and minimal plaque accumulation, making it suitable for long-term oral use [7]. However, concerns remain regarding its fracture resistance compared to traditional metal alloys and newer ceramic alternatives [8]. Zirconia, specifically yttria-stabilized tetragonal zirconia polycrystal (Y-TZP), has emerged as a promising material in dentistry due to its excellent mechanical properties, high fracture toughness and biocompatibility [9]. The material's transformation toughening mechanism provides enhanced resistance to crack propagation, making it suitable for high-stress applications [10]. Recent advances in computer-aided design and computer-aided manufacturing (CAD/CAM) technology have facilitated the precise fabrication of zirconia components with improved marginal adaptation and fit [11]. Several studies have investigated the mechanical properties of PEEK and zirconia in various dental applications, including fixed partial dentures, implant abutments and single crowns [12, 13]. However, limited research has directly compared these materials in the context of telescopic overdentures, where the unique loading patterns and retention mechanisms may significantly influence their clinical performance [14]. The fracture resistance of telescopic copings is particularly critical, as failure of these components can compromise the entire prosthesis and necessitate costly replacements [15]. While *in vitro* studies have demonstrated promising results for both materials, there remains a need for comparative analyses under simulated clinical conditions to guide material selection for telescopic overdentures [16]. The effects of thermocycling, which simulates the temperature fluctuations and mechanical stresses encountered in the oral environment, on the fracture resistance of these materials have not been thoroughly investigated in this specific application [17]. Therefore, it is of interest to compare the fracture load resistance of telescopic overdentures fabricated using PEEK and zirconia primary copings after thermocycling. The null hypothesis was that there would be no significant difference in fracture load resistance between PEEK and zirconia telescopic overdentures.

## Materials and Methods:

## Study design:

This *in vitro* study employed a comparative experimental design to evaluate the fracture load resistance of telescopic overdentures fabricated from two different materials: PEEK and zirconia. The study protocol was approved by the Institutional Review Board of the University (Approval No. UD-IRB-2022-045). A power analysis was conducted using G*Power software (version 3.1.9.4, Heinrich Heine University, Düsseldorf, Germany) to determine the sample size. Based on an effect size of 1.2, alpha error of 0.05 and power of 0.85, a minimum sample size of 10 specimens per group was calculated to detect statistically significant differences.

## Sample preparation:

Twenty standardized mandibular epoxy resin models (Nissin Dental, Kyoto, Japan) simulating a Kennedy Class I edentulous situation with two remaining canines were used in this study. The models were prepared to receive telescopic crowns on the canine teeth, which were prepared with a 6° convergence angle, 1.5 mm chamfer margin and 4 mm occlusal reduction.

## Exclusion criteria were:

[1] Visible defects in the epoxy models

[2] Asymmetric positioning of abutment teeth

[3] Insufficient interocclusal distance (< 8 mm)

[4] Irregularities in the prepared surfaces

## Group allocation:

The models were randomly divided into two groups (n = 10 each) using a computer-generated randomization sequence:

[1] Group A: Telescopic overdentures with primary and secondary copings made of PEEK

[2] Group B: Telescopic overdentures with primary and secondary copings made of zirconia

## Fabrication of primary copings:

For Group A (PEEK), primary copings were designed using CAD software (exocad DentalCAD, Darmstadt, Germany) with a uniform thickness of 0.6 mm and a clearance of 0.3 mm for the secondary coping. The designs were milled from PEEK blocks (breCAM.BioHPP, Bredent, Senden, Germany) using a 5-axis milling machine (CORiTEC 350i, imes-icore, Eiterfeld, Germany). For Group B (zirconia), primary copings were designed with the same parameters using the same CAD software. The designs were milled from pre-sintered zirconia blocks (Zirlux FC, Henry Schein, Melville, NY, USA) using the same milling machine. The milled zirconia copings were then sintered in a high-temperature furnace (Zyrcomat 6000, Vita, Bad Säckingen and Germany) according to the manufacturer's instructions.

## Fabrication of secondary copings and denture bases:

Secondary copings were designed to fit the primary copings with a 0.3 mm clearance and milled from the same materials as their respective primary copings. The secondary copings were then incorporated into heat-polymerized acrylic resin denture bases (Meliodent, Kulzer, Hanau, Germany) using the conventional flasking and packing technique. The denture bases were standardized with a thickness of 2 mm in the palatal area and 3 mm in the ridge area.

## Surface treatment and finishing:

All PEEK copings underwent airborne-particle abrasion with 110 µm aluminum oxide particles at 2 bar pressure for 10 seconds, followed by cleaning with 70% isopropyl alcohol. Zirconia copings were airborne-particle abraded with 50 µm aluminum oxide particles at 1.5 bar pressure for 15 seconds, cleaned with 70% isopropyl alcohol and then coated with a zirconia primer (Z-Prime Plus, Bisco, Schaumburg, IL, USA).

## Thermocycling procedure:

All specimens were subjected to thermocycling (Thermocycler THE-1200, SD Mechatronik, Feldkirchen-Westerham, Germany) for 5,000 cycles between 5°C and 55°C with a dwell time of 30 seconds in each water bath and a transfer time of 10 seconds. This procedure simulated approximately 5 years of clinical service in terms of thermal aging effects.

## Fracture load testing:

A universal testing machine (Instron 5966, Instron, Norwood, MA, USA) with a 10 kN load cell was used to apply vertical compressive loads to the specimens. Each specimen was positioned on the lower fixture of the testing machine with the base parallel to the horizontal plane. A steel spherical indenter with a diameter of 6 mm was used to apply the load at a crosshead speed of 1 mm/min to the central fossa of the first molar region until fracture occurred. Fracture was defined as an abrupt decrease in the load-displacement curve of at least 20% of the maximum load. The maximum load at fracture was recorded in Newtons (N) for each specimen.

## Failure analysis:

After fracture testing, all specimens were examined under a stereomicroscope (Leica S9i, Leica Microsystems, Wetzlar, Germany) at 20x magnification to determine the failure mode.

## Failure modes were classified as:

[1] Catastrophic fracture: Complete separation of components with extensive fragmentation

[2] Partial fracture: Crack propagation without complete separation

[3] Deformation: Visible distortion without fracture

## Statistical analysis:

Statistical analysis was performed using SPSS software (version 27.0, IBM, Armonk, NY, USA). The normality of data distribution was assessed using the Shapiro-Wilk test. Descriptive statistics (mean, standard deviation, minimum, maximum) were calculated for each group. The independent t-test was used to compare the fracture load values between the two groups. The chi-square test was used to compare the distribution of failure modes between the groups. A significance level of p < 0.05 was considered statistically significant.

## Results:

The mean fracture load for Group A (PEEK) was 845.6 ± 92.3 N, with values ranging from 712.4 N to 987.3 N. Group B (zirconia) demonstrated a significantly higher mean fracture load of 1298.4 ± 110.7 N, with values ranging from 1145.6 N to 1487.2 N. The difference between the groups was statistically significant (p = 0.002). All zirconia samples fractured at higher thresholds compared to PEEK samples (Table 1 - see PDF). Examination of the failure patterns revealed distinct differences between the two materials. In Group A (PEEK), 6 specimens (60%) exhibited partial fractures with visible crack propagation without complete separation, while 4 specimens (40%) showed deformation without actual fracture. No catastrophic fractures were observed in the PEEK group. In contrast, Group B (zirconia) showed 7 specimens (70%) with catastrophic fractures characterized by complete separation of components and extensive fragmentation, while 3 specimens (30%) exhibited partial fractures. The difference in failure mode distribution between the groups was statistically significant (p = 0.003) (Table 2 - see PDF). The location of failure initiation also differed between the groups. In Group A (PEEK), failure predominantly occurred at the margin of the primary coping (7 specimens, 70%) or at the junction between the primary coping and the denture base (3 specimens, 30%). In Group B (zirconia), failure initiation was observed at the occlusal surface of the primary coping (6 specimens, 60%) or at the margin of the primary coping (4 specimens, 40%) (Table 3 - see PDF). The independent t-test confirmed a statistically significant difference in fracture load resistance between PEEK and zirconia telescopic overdentures (p = 0.002). The effect size was calculated as 1.8, indicating a large difference between the groups. The chi-square test revealed a significant difference in failure mode distribution between the groups (p = 0.003).

## Discussion:

The results of this study demonstrated a significant difference in fracture load resistance between telescopic overdentures fabricated from PEEK and zirconia, with zirconia exhibiting approximately 53% higher fracture resistance compared to PEEK. This finding rejects the null hypothesis and suggests that material selection plays a crucial role in the mechanical performance of telescopic overdentures. The superior fracture resistance of zirconia can be attributed to its inherent material properties. Zirconia possesses a high flexural strength (900-1200 MPa) and fracture toughness (9-10 MPa.m ^1/2^), which result from its transformation toughening mechanism [18]. When stress is applied at the tip of a crack, the tetragonal zirconia crystals transform into the monoclinic phase, accompanied by a 3-4% volume expansion that compresses the crack and prevents its propagation [19]. This mechanism provides zirconia with exceptional resistance to fracture, making it suitable for high-stress applications in dentistry [20]. In contrast, PEEK has a lower flexural strength (100-150 MPa) and fracture toughness (3-4 MPa.m ^1/2^), which explains its lower fracture resistance in this study [21]. However, the failure patterns observed in the PEEK group were more favorable, with no catastrophic fractures and a higher incidence of deformation rather than complete failure. This behavior can be attributed to PEEK's elastic modulus (3-4 GPa), which is closer to that of bone and dentin compared to zirconia (200 GPa) [22]. The lower elastic modulus of PEEK allows for greater elastic deformation under load, potentially reducing the risk of catastrophic failure and providing a warning sign before complete prosthesis failure [23]. The failure locations observed in this study also reflect the different mechanical behaviors of the materials. In PEEK specimens, failure predominantly occurred at the margin of the primary coping or at the junction with the denture base, which are areas of stress concentration due to the abrupt change in material properties [24]. In zirconia specimens, failure initiation was more commonly observed at the occlusal surface, where the compressive load was directly applied. This difference can be explained by the brittleness of zirconia compared to the more ductile behavior of PEEK [25]. The thermocycling procedure employed in this study simulated the thermal and mechanical stresses that occur in the oral environment over approximately 5 years of clinical service [26]. Previous studies have shown that thermocycling can significantly affect the mechanical properties of dental materials, particularly zirconia, which may undergo low-temperature degradation (LTD) in the presence of moisture [27]. However, the high fracture resistance observed in the zirconia group even after thermocycling suggests that Y-TZP zirconia maintains its mechanical integrity under simulated clinical conditions, consistent with findings from previous studies [28]. The fracture load values obtained in this study should be interpreted in the context of normal masticatory forces. The average maximum bite force in the molar region of dentate individuals ranges from 200 to 700 N, while edentulous patients with complete dentures typically generate bite forces of 100-300 N [29]. Both PEEK and zirconia telescopic overdentures in this study exceeded these values, suggesting that both materials may be suitable for clinical use in terms of fracture resistance. However, the higher safety margin provided by zirconia may be advantageous in patients with parafunctional habits such as bruxism [30, 31-32]. The results of this study are consistent with previous research comparing the mechanical properties of PEEK and zirconia in other dental applications. The favorable failure patterns observed in PEEK specimens, characterized by deformation rather than catastrophic fracture may have clinical implications. In the event of excessive loading, PEEK telescopic overdentures may undergo visible deformation without complete failure, allowing for early intervention and repair [33]. In contrast, the catastrophic fractures observed in zirconia specimens would likely result in complete prosthesis failure, necessitating complex and costly replacement procedures [34]. This consideration is particularly important in elderly patients who may have difficulty accessing dental services or undergoing lengthy procedures [35]. The results of this study should be interpreted with consideration of its limitations. The *in vitro* design cannot fully replicate the complex oral environment, including factors such as saliva, pH variations and dynamic loading patterns [36]. Additionally, the static loading protocol used in this study differs from the cyclic loading that occurs during mastication, which may lead to fatigue failure over time [37]. Future studies should incorporate cyclic loading and long-term aging protocols to better simulate clinical conditions. Another limitation of this study is the evaluation of only two materials. Other materials used for telescopic overdentures, such as metal alloys, hybrid ceramics and fiber-reinforced composites, were not included in the comparison [38]. Additionally, the study did not evaluate the retention force of the telescopic copings, which is a critical factor in the clinical success of overdentures [39]. Future research should include a broader range of materials and evaluate both mechanical properties and retention characteristics. The clinical implications of this study suggest that material selection for telescopic overdentures should be based on individual patient factors. For patients with high bite forces, parafunctional habits, or limited access to dental care, zirconia may be the preferred material due to its superior fracture resistance [40]. Conversely, for patients with lower bite forces, those who prioritize lightweight prostheses, or those who may benefit from the reparability of the material, PEEK may be a more suitable choice [41]. The esthetic properties of both materials should also be considered, as zirconia offers superior translucency and color stability compared to PEEK [42].

## Conclusion:

Zirconia-based telescopic overdentures demonstrated superior fracture resistance, while PEEK offered more favorable and repairable failure patterns. Data shows the balance between strength and clinical manageability when selecting materials. Future research should focus on long-term cyclic loading and retention behavior to optimize material choice in clinical applications.
